# Direct Inhibition of TNF-α Promoter Activity by Fanconi Anemia Protein FANCD2

**DOI:** 10.1371/journal.pone.0023324

**Published:** 2011-08-31

**Authors:** Nobuko Matsushita, Yujiro Endo, Koichi Sato, Hitoshi Kurumizaka, Takayuki Yamashita, Minoru Takata, Shigeru Yanagi

**Affiliations:** 1 Laboratory of Molecular Biochemistry, School of Life Science, Tokyo University of Pharmacy and Life Science, Hachioji, Tokyo, Japan; 2 Laboratory of Structural Biology, Graduate School of Advanced Science and Engineering, Waseda University, Shinjuku-ku, Tokyo Japan; 3 Laboratory of DNA Damage Signaling, Department of Late Effects Studies, Radiation Biology Center, Kyoto University, Kyoto, Kyoto, Japan; 4 Laboratory of Molecular Genetics, The Institute for Molecular and Cellular Regulation, Gunma University, Maebashi, Gunma, Japan; Emory University School of Medicine, United States of America

## Abstract

Fanconi anemia (FA), an inherited disease, is associated with progressive bone marrow failure, predisposition to cancer, and genomic instability. Genes corresponding to 15 identified FA complementation groups have been cloned, and each gene product functions in the response to DNA damage induced by cross-linking agents and/or in protection against genome instability. Interestingly, overproduction of inflammatory cytokines such as tumor necrosis factor alpha (TNF-α) and aberrant activation of NF-κB-dependent transcriptional activity have been observed in FA cells. Here we demonstrated that FANCD2 protein inhibits NF-κB activity in its monoubiquitination-dependent manner. Furthermore, we detected a specific association between FANCD2 and an NF-κB consensus element in the TNF-α promoter by electrophoretic mobility shift assays (EMSA) and chromatin immunoprecipitation (ChIP) assay. Therefore, we propose FANCD2 deficiency promotes transcriptional activity of the TNF-α promoter and induces overproduction of TNF-which then sustains prolonged inflammatory responses. These results also suggest that artificial modulation of TNFα production could be a promising therapeutic approach to FA.

## Introduction

Fanconi anemia (FA) is a genetic disorder associated with genome instability and mainly characterized by progressive bone marrow failure, congenital abnormalities, and predisposition to cancer[1], [2]. To date, 15 FA gene products (FANCA, B, C, D1, D2, E, F, G, I, J, L, M, N, O and P) have been identified and they constitute the FANC pathway, which is thought to function in preventing genome instability[1], [2], [3], [4]. The FA core complex comprises FAAP24, FAAP100, and 8 FA proteins (FANCA, B, C, E, F, G, L, and M) and mediates DNA-damage-induced or replication-stress-induced monoubiquitylation of FANCD2 and FANCI[1]. Monoubiquitinated FANCD2 and FANCI translocate to chromatin and function in DNA repair at least partially by recruitment of FAN1 nuclease[5], [6], [7].

Defective self-renewal of hematopoietic stem cells causes bone marrow failure, and its consequences (e.g. pancytopenia or myeloid malignancies) are the major cause of morbidity in FA patients[7]. Two different mechanisms, which are not necessarily mutually exclusive, may contribute to the development of the bone marrow failure in FA. First, DNA repair function of the FANC pathway seems necessary to maintain hematopoietic stem cell, and the compromised DNA repair activity in FA patients results in the accumulation of unrepaired DNA, leading to genome instability and depletion of functional hematopoietic stem cells[1], [8]. Second, it has been suggested that hematopoietic disorders in FA patients may result from hypersensitivity to cytokines, such as TNF-α; for example, cells lacking FANCC, a core complex component, are hypersensitive to the apoptotic effect of a pro-inflammation cytokine, TNF-α[9], [10], [11], [12], [13], [14]. Furthermore, abnormally elevated levels of serum and intracellular TNF-α have been reported in FA patients [15], [16]. Consistent with this, in FANCC-deficient murine hematopoietic stem cells, TNF-αoverproduction results in bone marrow hypoplasia, and long-term exposure of these cells to TNF-α induces clonal evolution that leads to myelogenous leukemia[13], [17]. The possibility of clinical trial of anti-TNF-α agents for the treatment of selected FA patients has been proposed [18]. However, definitive evidence for functional crosstalk between other FA proteins, such as FANCD2, and cytokine response/overproduction is lacking.

In this work, we identified that direct association of FANCD2 and NF-κB consensus sequence (κB1site) in TNF-αpromoter, leading to the repression of its transcriptional activity. Thus FANCD2 deficiency triggered TNF-α overproduction, which is reportedly a major cause of morbidity in FA mutant mice[17], [19].

## Results

### FANCD2 deficiency enhances TNF-α-induced NF-κB-dependent transcriptional activity

**Figure 1:**
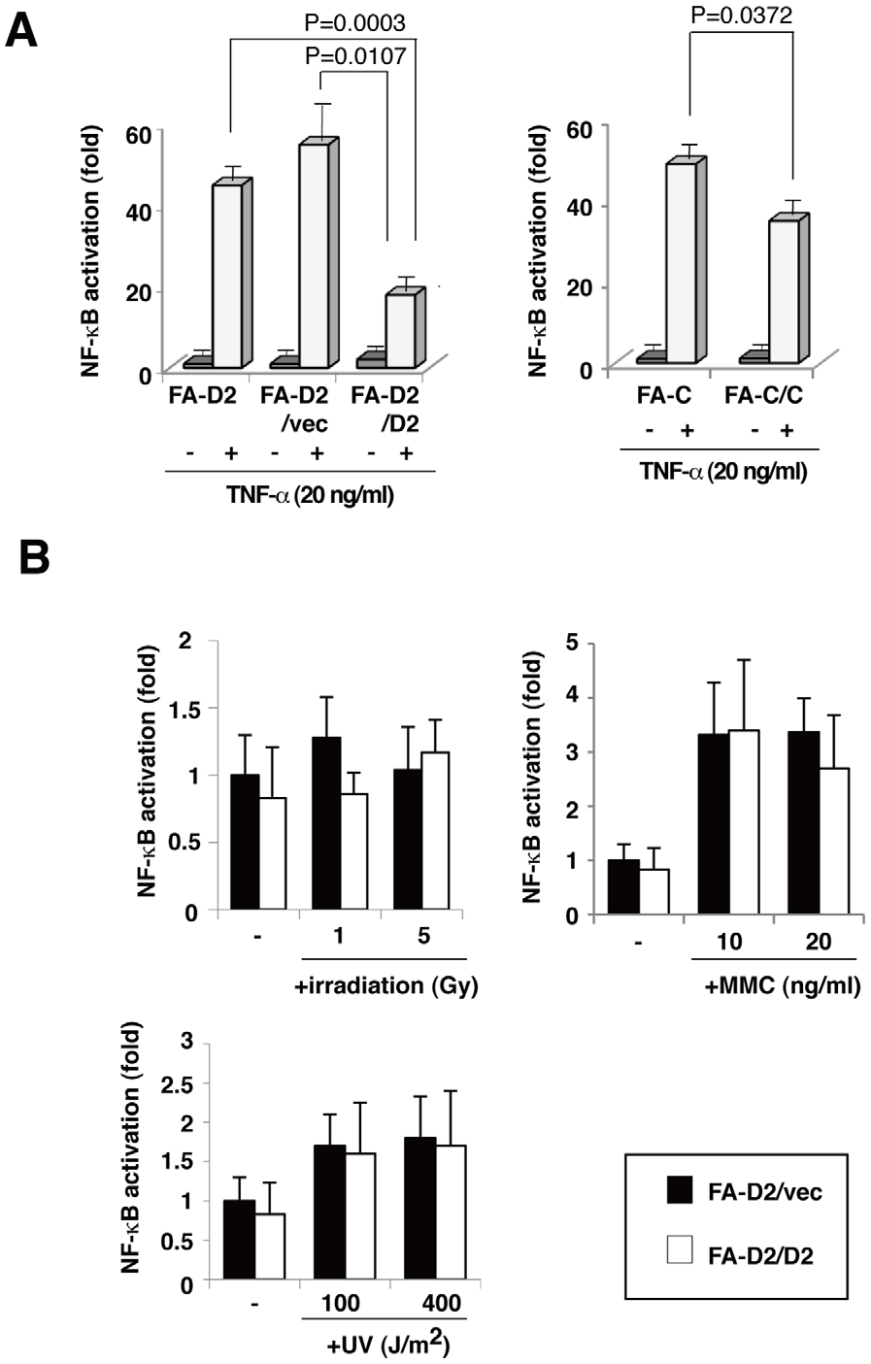
Deficiency of FANCD2 increases TNF-α-induced NF-κB-dependent transcriptional activity. (A) FA fibroblast cells derived from complementation group C and group D2 patients were designated FA-C (PD331) and FA-D2 (PD20), respectively. FA-C/C (FA-C; (PD331)+FANCC) and FA-D2/ D2 (FA-D2; (PD20)+FANCD2) were retrovirus-transformed derivatives of PD331 and PD20 that expressed functional FANCC and FANCD2, respectively. FA-D2/vec cells (FA-D2 expressing empty vector) were FANCD2-deficient derivatives of PD20. FA-D2 cells (FA-D2 and FA-D2/vec) showed higher TNF-α-induced NF-κB-dependent transcriptional activity than did the FANCD2-expressing cells (FA-D2/D2); similarly FANCC-deficient cells (FA-C) exhibited more NF-κB-dependent transcriptional activity than did FANCC-expressing cells (FA-C/C). FA fibroblasts were transfected with pNFκB-Luc (100 ng) and pRL-CMV (10 ng). Cells were treated with or without TNF-α (20 ng/ml) for 8 h. Cells were harvested and dual luciferase assays were performed. Fold activation represents the mean (± s.d) luciferase values of indicated cells normalized with respect to unstimulated FA-D2 cells, from three independent experiments. (B) FANCD2 did not have a significant effect on irradiation-, MMC- or UV-induced NF-κB activation. FA-D2 and FA-D2/D2 fibroblast cells were transfected with pNFκB-Luc (100 ng) and pRL-CMV (10 ng). Cells were treated with irradiation (1. 5 Gy), MMC (10, 20 ng/ml) or UV (100, 400 J/m^2^). Fold activation represents the mean (± s.d) luciferase values of indicated cells normalized with respect to un-stimulated FA-D2/vec cells, from three independent experiments.

TNF-α triggers several signaling pathways that converge on the activation of NF-κB, a transcription factor that is constitutively activated in FA cells and FANCC knockdown cells activated by TLR8 agonists[20], [21], [22]. We examined the role of FA proteins in NF-κB-dependent transcriptional activity induced by TNF-α. We compared three type of cells—a patient-derived FANCD2 mutant fibroblast cell line PD20 (FA-D2), PD20 cells complemented with a retrovirus containing the functional human FANCD2 cDNA (FA-D2/D2), and PD20 stably transduced with an empty vector (FA-D2/vec). We also included a patient-derived FANCC-/- fibroblast cell line PD331 (FA-C) and its derivative retrovirally transduced with FANCC (FA-C/C). All of these cells were transiently transfected with an NF-κB-dependent luciferase reporter plasmid containing four copies of the NF-κB consensus sequences (pNFκB-Luc). TNF-α-induced activation of NF-κB was higher in FANCD2-deficient cells (FA-D2, FA-D2/vec) than in FANCD2-proficient cells (FA-D2/D2); similarly, FANCC-deficient cells (FA-C) had higher levels of TNF-α-induced NF-κB activation than did the FANCC-proficient cells (FA-C/FANCC)(Fig 1A) We also showed that transiently expression of FANCD2WT repressed enhanced NF-κB transcriptional activity of FANCD2-deficient cells (FA-D2/vec). However, mutant FANCD2 (FANCD2K561R; a missense substitution at monoubiquitination site (K561)) and FANCC did not repress (Figure S1A). Moreover, there was not significant differences in TNF-α-induced NF-κB activation between FANCA-deficient cells (FA-A) and FANCA-proficient cells (FA-A/FANCA) (Fig. S1B).These data suggested that NF-κB transcriptional activity was influenced by FANCC and FANCD2. Several DNA-damaging agents that induce DNA double-strand breaks (e.g., ionizing radiation (IR)) elicit NF-κB-dependent transcription by activating ataxia telangiectasia-mutated kinase (ATM)[23]. MMC, a chemotherapeutic drug that induces formation of intra- and inter-strand DNA crosslinks, and UV also activate NF-κB[24], [25]. Therefore, we assessed the functional effect of FANCD2 on NF-κB following X-ray, UV, or MMC treatment; the FANCD2 deficiency did not affect the NF-κB-dependent transcriptional activity induced by any of these DNA-damaging agents (Fig. 1B). These data suggested that NF-κB transcriptional activity induced by mechanisms other than TNF-α was not significantly enhanced in FANCD2-deficient cells.

### FANCD2-deficient fibroblast cells were more sensitive to TNF-α

**Figure 2:**
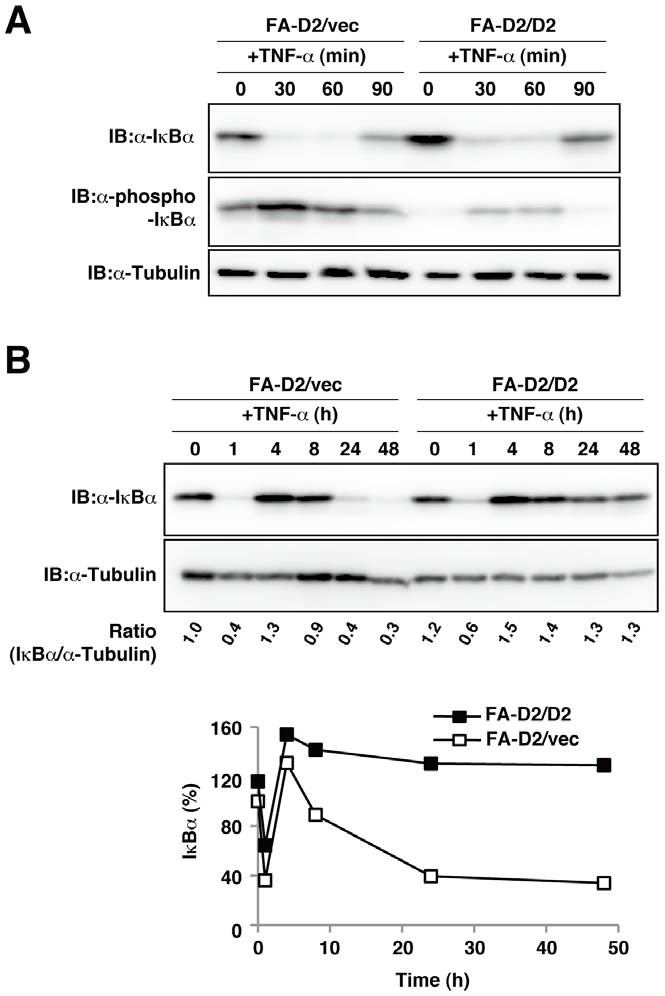
FANCD2-deficient fibroblast cells showed increased sensitivity to TNF-α. (A) FA-D2/vec and FA-D2/D2 fibroblast cells were stimulated for indicated time at 37°C with TNF-α(100 ng/ml), and cell lysates were examined by immunoblotting (IB) using anti-phospho (P) Ser 32/36 IκBα or anti-α-tubulin. FA-D2 fibroblast cells showed a higher NF-κB response to TNF-α than did FA-D2/D2 cells. (B) FA-D2/vec and FA-D2/D2 fibroblast cells were cultured for indicated times with TNF-α5ng/ml). Exposure to TNF-αinduced significantly decreased the amount of proteins in FA-D2/vec cells. The amount of IκB-α protein was represented as the ratio of IκB-α protein to α-tubulin and normalized with respect to unstimulated FA-D2/vec cells.

**Figure 3:**
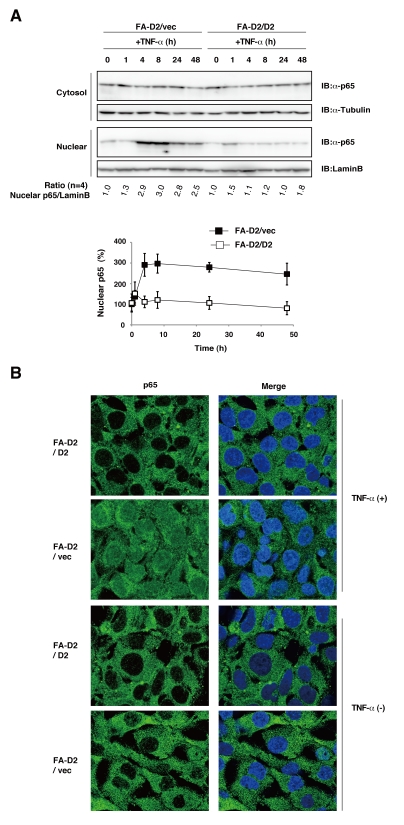
Enhanced TNF-α-induced p65/RelA nuclear translocation in FANCD2-deficient fibroblast cells. (A) FANCD2 deficiency enhanced TNF-α-induced nuclear localization of p65. FA-D2/vec and FA-D2/D2 fibroblast cells were cultured for indicated times with TNF-α5ng/ml). Cytoplasmic and nuclear extracts were prepared as described in “Materials and Methods” for immunoblot analysis. The amount of nuclear p65 protein was represented as the ratio of nuclear p65 protein to LaminBand normalized with respect to unstimulated FA-D2/vec cells. (B) Nuclear translocation of p65 was increased in FA-D2/vec cells. FA-D2/vec and FA-D2/D2 fibroblast cells were cultured for 24 h with or without TNF-α5ng/ml) and immunostained with anti-p65 antibody (green). Nuclei were stained with Hoechst (blue in Merge).

Pro-inflammatory cytokines inactivate the inhibitor protein IκBα, which sequesters NF-κB proteins in the cytoplasm. The multimeric IκB kinase (IKK) complex phosphorylates IκBα, resulting in the ubiquitination and degradation of IκBα, which in turn cause the release and nuclear translocation of NF-κB[26]. IκBα is an NF-κB target gene, and re-synthesis of IκBα terminates NF-κB activity by its nuclear export[27]. To confirm that the FANCD2 deficiency enhanced NF-κB activity, we quantified IκBα phosphorylation and the subsequent IκBα degradation in FANCD2-deficient and FANCD2-proficient cells. FANCD2-deficient cells (FA-D2/vec) treated with or without TNF-α showed significantly enhanced IκBα phosphorylation and decreased amounts of IκBα relative to complemented FANCD2 cells (FA-D2/D2) (Fig. 2A). We also examined the termination of TNF-α induced NF-κB activity in FA cells, using IκBα levels as a marker. The amount of IκBαprotein was represented as the ratio of IκBα protein to α-tubulin and normalized with respect to unstimulated FA-D2/vec cells. A 4-h exposure to TNF-αinduced transient loss and subsequent resynthesis of IκBα protein in FANCD2-deficient (FA-D2/vec) and FANCD2-proficient cells (FA-D2/D2) (Fig. 2B). However, after 24-h TNF-α exposure, the amount of IκBα protein was lower and the reduction was prolonged in FANCD2-deficient cells relative to FANCD2-proficient cells (Fig. 2B). We also observed that the TNF-α induced nuclear translocation of RelA/p65, which is one of the dimeric NF-κB transcriptional complex, was significantly higher in FANCD2-deficient cells than in control cells (Fig. 3A, B). Collectively, these data suggested that FANCD2 has a negative regulatory function in TNF-α-induced NF-κB activation.

### Inhibition of NF-κB activity and expression of TNF-α mRNA by FANCD2

**Figure 4:**
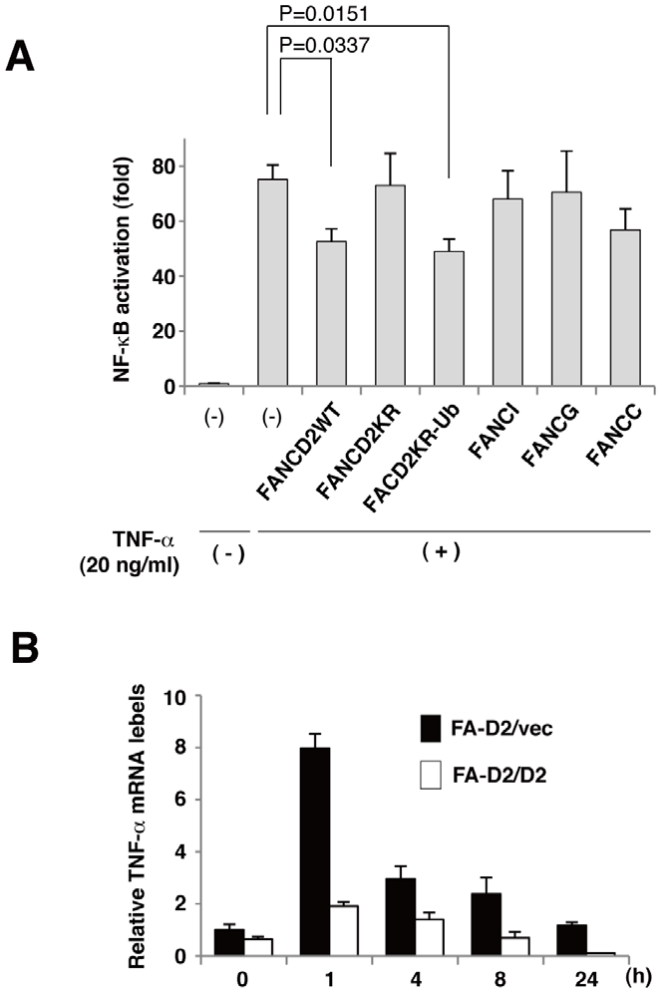
FANCD2 negatively regulates NF-κB activity and TNF-α transcription. (A) Overexpression of FANCD2 WT or FANCD2KR-Ub (FANCD2 K561R-Ub fusion), but not the K561R mutant (FANCD2KR), inhibited NF-κB-dependent transcriptional activity. HEK293 cells were transfected with FANCD2 WT, FANCI, FANCG, FANCC, FANCD2 KR, or FANCD2 KR-Ub (each 100 ng) and pNFκB-Luc (100 ng) and pRL-CMV (10 ng). Cells were treated with or without TNF-α(20 ng/ml) for 8 h. Cells were harvested, and dual luciferase assays were performed. Fold activation represents the mean (± s.d) luciferase values of TNF-αstimulated cells normalized with respect to unstimulated cells, from three independent experiments. (B) FA-D2/vec and FA-D2/D2 fibroblast cells were treated with TNF-α (5 ng/ml) for the indicated periods, and real-time PCR analysis of TNF-α mRNA expression in these cells was performed. Gene expression results represent four independent experiments, normalized with respect to unstimulated FA-D2/vec cells, mean (± s.d) from triplicate values are shown.

To test the ability of FANCD2 and other FA proteins to modulate NF-κB-dependent transcriptional activity, HEK293 cells were transfected with an NF-κB-dependent luciferase reporter plasmid (pNFκB-Luc) and FA protein cDNA. As expected, overexpression of FANCD2 inhibited TNF-α-induced NF-κB-dependent transcriptional activity. However, FANCG, FANCI and a mutant FANCD2 (FANCD2K561R (K561)) did not inhibit NF-κB activity. FANCC also did not significantly inhibit (p = 0.09739). In addition, we used a FANCD2 derivative FANCD2KR fused with a single ubiquitin moiety (FANCD2K561R-Ub), which was previously shown to restore near wild-type levels of cisplatin tolerance in FANCD2-deficient cells[28]. We found that TNF-α-induced NF-κB-dependent transcriptional activity was similar in FANCD2KR-Ub-expressing and FANCD2-expressing cells (Fig. 4A). As a positive control, we confirmed that knockdown of endogeneous p65 with a pool of four distinct siRNA duplexes repressed TNF-αinduced NF-κB activation compared with control scrambled siRNA (Fig.S2). These data indicated that FANCD2 negatively regulated NF-κB transcriptional activity in a monoubiquitination dependent manner. Following TNF-α stimulation, TNF-α transcript levels were significantly higher in FANCD2-deficient cells than in FANCD2-proficient cells (Fig. 4B). Collectively, these data indicate that FANCD2 suppresses NF-κB transcriptional activity, and that FANCD2 deficiency enhanced expression of TNF-αmRNA.

### FANCD2 represses TNF-α promoter activity

**Figure 5:**
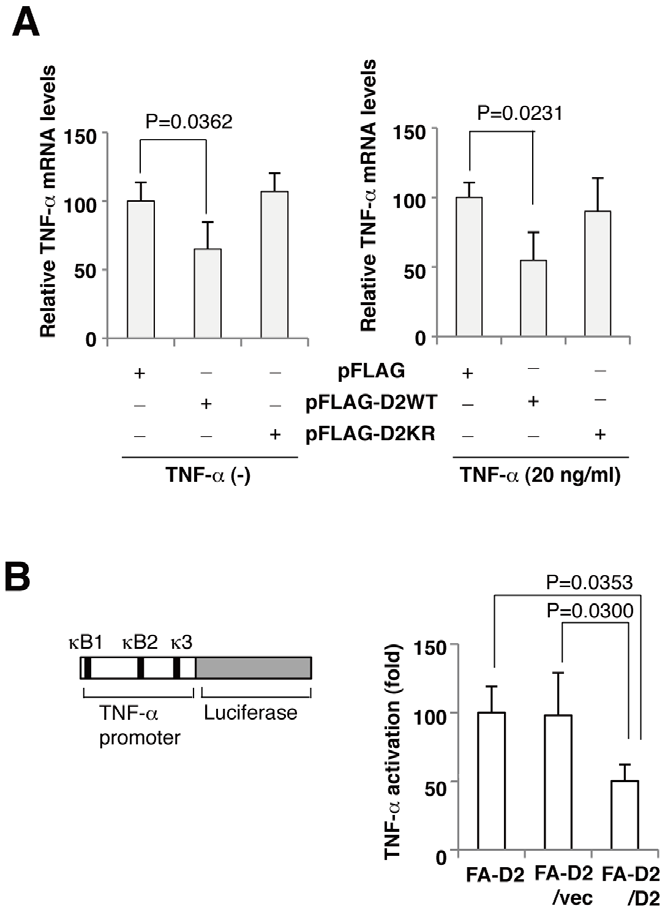
Disruption of FACD2 enhances promoter activity of TNF-α gene. (A) Real-time PCR analysis of TNF-α mRNA expression in FA-D2 (PD20) cells, after transfection with FANCD2 WT, FANCD2 K561R, or empty vector. Cells were treated with or without TNF-α(20 ng/ml). Overexpression of FANCD2 WT, but not FANCD2 K561R, could inhibit TNF-α mRNA expression. Results represent three independent experiments, normalized with respect to those obtained from cells expressing empty vector, shown are the mean (± s.d) from triplicate experiments. (B) Schematic representation of the TNF-α promoter/reporter gene construct. FA-D2 cells (FA-D2, FA-D2/vec cells) showed higher TNF-α-induced TNF-α transcriptional activity than FANCD2-expressing cells (FA-D2/D2). Fold activation represents the mean (± s.d) luciferase values of TNF-α-stimulated cells normalized with respect to FA-D2 cells from three independent experiments.

To assess whether FANCD2 could directly inhibit the expression of TNF-α, FANCD2-deficient cells were transfected with FANCD2 or FANCD2KR cDNA, and relative TNF-α mRNA levels were quantified. The elevated expression of TNF-αinFANCD2-deficient cells was decreased when FANCD2, but not FANCD2KR, was transiently expressed with or without TNF-α (Fig. 5A). Next, we examined the regulation of TNF-α promoter activity by FANCD2. FANCD2-deficient cells (FA-D2/vec) and FANCD2-expressing cells (FA-D2/D2) were transfected with a reporter plasmid (pTNF-ãluc expressing firefly luciferase under the control of the human TNF-α promoter (Fig. 5B). Relative TNF-α promoter activity was significantly higher in FANCD2-deficient cells than in FANCD2-proficient cells (Fig. 5B). These results suggested that FANCD2 directly inhibit TNF-α transcription by repressing TNF-α promoter activity.

### FANCD2 directly binds to NF-κB consensus in TNF-α promoter

**Figure 6:**
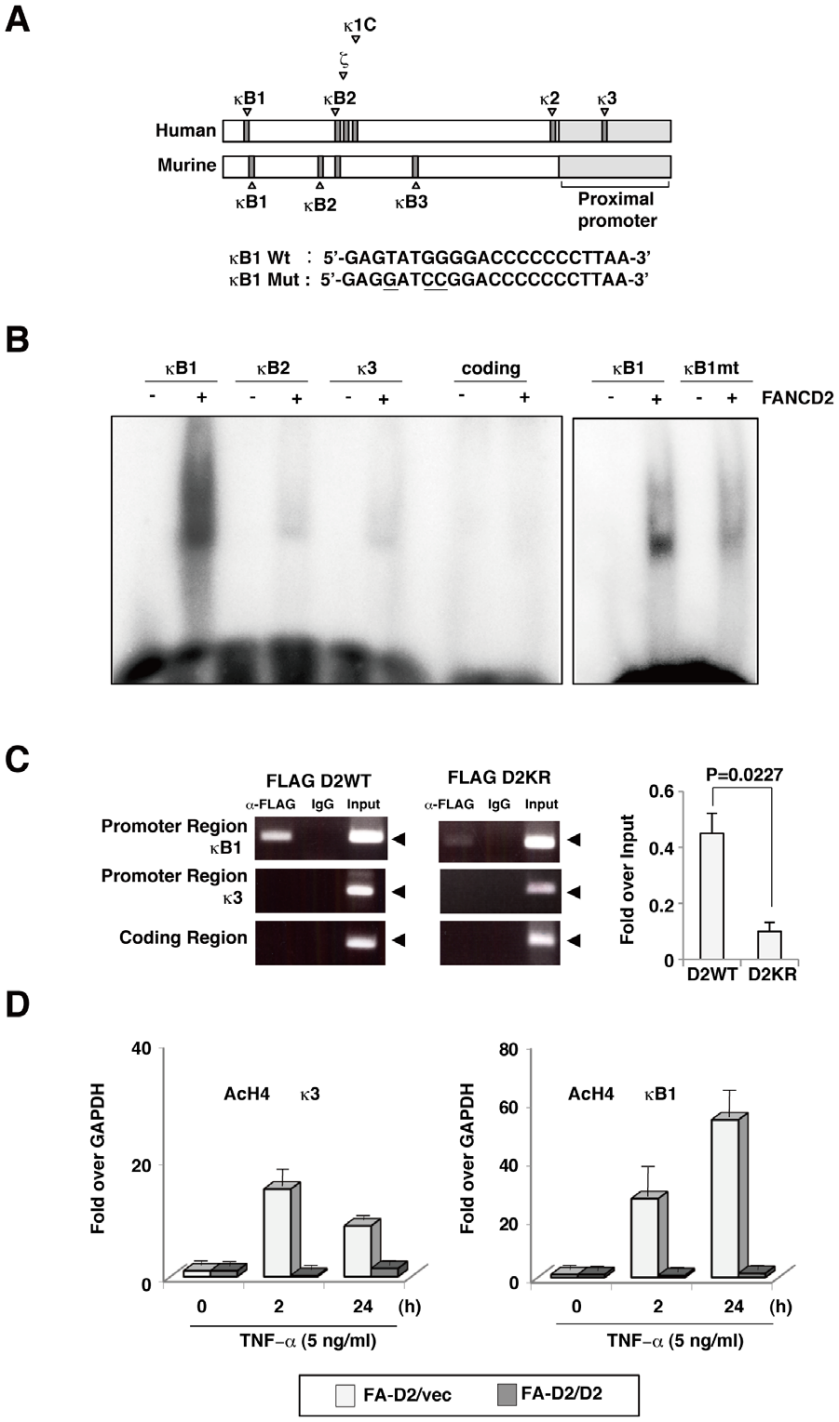
FANCD2 directly binds to the TNF-α promoter. (A) Schematic representation of the promoter region of the TNF-α gene and three NF-κB consensus sites (κB1, κB2, κ3)[34] used in gel-shift assays; site-directed mutations at κB1 are shown. (B) EMSA assay demonstrated that FANCD2 bound strongly and specifically to an NF-κB binding site previously described in the TNF-α genomic sequence. Site-directed mutation at κB1 affected FANCD2 binding based on the EMSA assay. (C) FANCD2 associated with the promoter region of TNF-α but not with the coding region. Lysates of HeLa cells expressing FLAG-FANCD2WT or FLAG-FANCD2K561R were subjected to ChIP with indicated antibodies (FLAG antibody) followed by PCR amplification of the TNF-α promoter (κB1 or κB3) or coding DNA sequence as indicated in manual (EZ CHIP, upstate). Immunoprecipitated DNA and input DNA were analyzed by qPCR amplification of TNF-α promoter (κB1) and GAPDH sequences, and represented as % input (κB1) / % input (GAPDH). Results represent three independent experiments, the mean (± s.d) from triplicated values are shown. (D) FA-D2 and FA-D2+D2 cells were stimulated with TNF-α for the indicated periods and analyzed by ChIP (AcH4). Immunoprecipitated DNA and input DNA were analyzed by qPCR amplification of NF-κB promoter (κB1 or κB3) sequences as indicated. ChIP data are presented as %input (gene specific)/%input (GAPDH) and were normalized with respect to those obtained in unstimulated FA-D2/vec cells. Results represent three independent experiments; means (± s.d) from triplicate values are shown.

Because TNF-α plays an important role in diverse cellular events such as induction of other cytokines, cell proliferation, differentiation, and apoptosis[29], the regulation of TNF-α expression is tightly controlled by several transcription factors, including NF-κB[30], NF-AT[31], or activating protein I (AP-1)[32]. For example, multiple NF-κB/Rel-binding sites in the mouse TNF-α promoter contribute to the TNF-α transcriptional response to lipopolysaccharide (LPS)[30], and these binding sites are designated as κB1, κB2kB2and kB3. NF-κB-like sites (kB1, κB2 ζ κ1, k2 and k3 in the human TNF-α promoter have been identified[33] Fig. 6A Of these NF-κB-like sites, κB1 apparently has the highest affinity for NF-κB/Rel proteins[34]. To investigate how FANCD2 suppresses TNF-α promoter activity, we performed electrophoretic mobility shift assays (EMSA) with the recombinant FANCD2 protein and oligonucleotides derived from the κB1, κB2 and κ3 sites in the human TNF-α promoter or sequences from the coding regions of the TNF-α gene. Stronger binding to FANCD2 was observed with the κB1oligonucleotide than with the kB2 or k3 oligonucleotide, while FANCD2 could not bind to the oligonucleotide from coding regions (Fig. 6B. Moreover, a mutation in the κB1 oligonucleotide (Fig. 6A significantly reduced its FANCD2 binding (Fig. 6B. In addition, we also performed EMSA, using labeled κB1 oligonucleotide and 2 µg of nuclear extracts from HEK293 cells transfected with empty vector, Flag-tagged FANCD2WT, FANCD2KR, or FANCD2KR-Ub. FANCD2WT, FANCD2KR and FANCD2KR-Ub display similar κB1 oligonucleotide binding activity. These results showed that FANCD2 binds to κB1 oligonucleotide sequence specifically. However, monoubiquitination is not required for FANCD2 binding to the κB1site oligonucleotide. FANCD2 was reported to bind to double stranded DNA ends and Holliday junctions[35]; moreover, sequence specific binding of FANCD2 at telomeric sequences has been reported [36]. Thus, these data demonstrated that FANCD2 binds to some specific DNA sequences.

To investigate whether FANCD2 was recruited to the NF-κB-like site in the TNF-α promoter, we employed a chromatin immunoprecipitation (ChIP) assay. HeLa cells were transfected with Flag-tagged wild-type FANCD2 or Flag-tagged monoubiquitination site mutant FANCD2 (FANCD2KR), the ChIP assays were performed using anti-Flag antibodies. PCR primers were designed to amplify a proximal or a distal region of the TNF-α promoter, containing the κB1or κ3 site, respectively, or to amplify a coding region in TNF-α. FANCD2WT bound to the distal region containing the κB1site with higher affinity than did FANCD2KR, and neither FANCD2WT nor FANCD2KR bound the proximal promoter region or the coding regions (Fig. 6C). Post-translational modifications of histones regulate chromatin structure and the dynamics of transcription. Acetylation of histone H4 is a prevalent and reversible modification that is used as a marker of transcriptional activation[37]. We performed ChIP assays using antibodies recognizing acetylated histone H4. We found that the κB1andk3sites of the TNF-α promoter in FANCD2-deficient cells contained acetylated histone H4 at 2 h or 24 h after TNF-αstimulation, whereas in FANCD2-proficient cells the histone H4 at these sites were not detectably acetylated (Fig. 6D). These data suggested that FANCD2 deficiency significantly enhanced TNF-α promoter activity, and resulted in TNF-α overproduction.

## Discussion

FA proteins functions in repair or bypass of interstrand cross-links (ICLs) during replication, and the hallmark of FA-deficient cells is hypersensitivity to ICLs that accompany chromosome aberrations[1], [8]. FA-deficient cells also overproduce TNF-α[15], [16]. Moreover, in FANCC-deficient murine hematopoietic stem cells, overproduction of and hypersensitivity to TNF-α results in bone marrow hypoplasia and long-term exposure of these cells to TNF-α induced clonal evolution that led to myelogenous leukemia[13], [17]. However, how disruption of the other FA proteins, such as FANCD2, up-regulate production of TNF-α and results in chronic inflammation has not been addressed.

In this work, we showed that FANCD2 directly bound to the distal region of the TNF-α promoter which contains an NF-κB consensus sequence (κB1 site), leading to repression of its transcriptional activity. In addition, we demonstrated that FANCD2 negatively regulated NF-κB transcriptional activity in a monoubiquitination-dependent manner (Fig. 4A), and the monoubiquitiantation site mutant of FANCD2 could not be recruited to the κB1 site of TNF-α promoter region (Fig. 6C). However, monoubiquitination itself is not required for FANCD2 binding to the κB1 site oligonucleotide (Fig. 6B, S3). We previously showed that monoubiquitination of FANCD2 is required for targeting of FANCD2 to chromatin [28]. These results suggest that monoubiquitination is essential for FANCD2 to translocate to chromatin for binding to the TNF-αpromoter region in transcriptional activity. Although the precise mechanism is still unclear, FANCD2 deficiency enhanced histone acetylation of TNF-αpromoter region and increased TNF-α mRNA (Fig. 4B, 6D), resulted in overproduction of TNF-α observed in FA patients. Histone acetylation is a positive mark associated with transcriptionally active chromatin, whereas deacetylated histones are found in closed, inactive chromatin[38]. These data suggested that FANCD2 induces TNF-α gene specific regulation at the level of chromatin including nucleosome remodeling and covalent histone modifications.

These data unravel the molecular links connecting disruption of the FANCD2 with increased inflammation due to TNF-αoverproduction. Our results clearly indicated that FANCD2 directly represses an immune response by regulating the transcriptional activity of an inflammatory mediator, TNF-α. These observations established a new function for the FANCD2 in addition to its well-documented role in preventing genome instability as a genome caretaker. The disruption of these two functions might have a significant impact on morbidity in FA patients. Thus, our study indicates a mechanism that links the FA protein FANCD2 with the inflammation response.

## Materials and Methods

### Reagents

Recombinant human TNF-α (210-TA) was purchased from R&D systems. MMC was purchased from Kyowa Hakkou (Tokyo, Japan). Antibody to IκBα (4814) and Phospho-IκBα (9246) were purchased from Cell Signaling. Antibody to H4-Ac (06-866) was purchased from Millipore. Antibodies to p65/RelA (sc-109) and Lamin B (sc-6217) were purchased from Santa Cruz Biotechnologies. Antibodies to FLAG (F3165) and α-Tubulin (T9026) were purchased from SIGMA.

### Plasmids and siRNA

pNFκB-Luc was purchased from Clontech (Mountain View, CA). pTNF-αwt-luc was generated by Human TNF-α promoter sequence[34] (21173 to 1130 nt) PCR amplified with primers (5′-cggggtaccGAGGGACAGAGGGCTCAAAGG-3′, 5′-cggaagcttGGAAGAGAACCTGCCTGGCAG-3′) from 293 cells, digested with Kpn1 and HindIII, and subcloned into pGL4.14. pTNF-ακB1-mt-luc was generated by site-directed mutagenesis at site κB1 with primers (5′-GAGTATGGGGACCCCCCCTTAA-3′, 5′-TTAAGGGGGGGTCCCCATACTC-3′) using pTNF-αwt-luc as a template. All constructs were verified by DNA sequencing. Control siRNA and human p65 siRNA on TARGET SMART pool were purchased from Dharmacon. Transfect reagents used was DharmaFECT (Dharmacon).

### Cell culture conditions and luciferase assays

SV-40 transformed FA-D2 fibroblasts PD20 (FA-D2), PD20 expressing empty vector (FA-D2/vec), PD20 complemented with human FANCD2 (FA-D2/D2), FA-C fibroblasts PD331 (FA-C), PD331 complemented with human FANCC (FA-C/C) were gifts from Dr. Fang Zhang (Fanconi Anemia Cell Repository, The Oregon Health and Science University). GM6914 FANCA-null fibroblasts (FA-A) and GM6914 complemented with human FANCA (FA-A/A) were gifts from Dr. Takayuki Yamashita (Gunma University). These cells were cultured at 37°C with 5% CO2 using a-minimal essential medium supplemented with 20% fetal calf serum. HeLa cells and HEK293 cells were from ATCC. These cells were grown in DMEM medium supplemented with 10% fetal calf serum at 37°C with 5% CO2. For luciferase assays, cells (85–90 % confluency in 96-well plates) were transiently transfected with 100 ng of indicated luciferase reporter plasmid (pNF-κB-Luc, pTNF-αwtLuc and pTNF-α mtLuc and 10 ng of phRL-TK (Promega, Madison, WI, USA) using LipofectAMINE 2000 regent (Invitrogen), following the manufacturer's protocol. Cells were harvested 24 h after transfection and incubated with or without indicated reagents (TNF-αMMC) for 8–24 h, and cell lysates were assayed for luciferase activity using the dual luciferase assay system (Promega, Madison, WI, USA). *Firefly* luciferase activity was corrected for transfection efficiency using the *Renilla* luciferase activity. Results presented are the average of triplicate experiments with the S.D. values shown as error bars. P-value was calculated from three independent experiments using a t-test.

### Subcellular fractionation and detection of cytoplasmic or nuclear NF-κB

For fractionation experiments, cells incubated with TNF-α5 ng/ml) for indicated time were collected by centrifugation and washed in PBS. The cell pellet containing 5×10^6^ cells were suspended in 100 µL hypotonic buffer (50 mM Tris [pH 7.4], 10 mM NaCl, 5 mM EDTA, 0.05% Nonidet P-40 [NP-40]) containing protease inhibitor, 1 mM NaF, and 1 mM Na3VO4. After 10 minutes on ice, the lysate was centrifuged for 10 min at 500 g at 4°C, and the supernatant was collected as cytoplasmic lysates. The pellet was washed 5 times in hypotonic buffer containing 0.1% NP-40, and the remaining pellet was suspended in 100 µL RIPA buffer (20 mM Tris-HCl (pH 7.5), 150 mM NaCl, 1 mM Na2EDTA, 1 mM EGT, 1% NP-40, 1% sodium deoxycholate, 2.5 mM sodium pyrophosphate, 1 mM beta-glycerophosphate, 1 mM Na3VO4, 1 µg/ml leupeptin) containing protease and phosphatase inhibitor. After 10 minutes, the lysate was centrifuged at 20,000 g for 15 minutes, and the supernatants was collected as nuclear lysates[39].

### Western blotting analysis

Whole-cell lysates (prepared with SDS sample buffer), cytoplasmic or nuclear fractions were separated with polyacrylamide gel, transferred to a polyvinylidene difluoride membranes (Millipore), and detected with anti human IκBα, Phospho-IκBα, p65/RelA, Lamin B or α-Tubulin antibodies and ECL reagents (GE Healthcare, Piscataway, New Jersey).

### Immunofluorescence

Cells were fixed with 4% paraformaldehyde for 10 minutes, followed by 6 min permeabilization in Triton buffer (0.5% Triton X-100 in PBS) at room temperature. Coverslips were blocked with 5% BSA in PBS and then incubated with primary antibody to RelA/p65 for 1 hour. Cells were washed and incubated with secondary antibody conjugated to Alexa Fluor 488 (Molecular Probes). DNA was stained with Hoechst (Sigma). Fluorescence microscopy was performed on a FV1000 microscope (Olympus). Images were then imported in Adobe Photoshop Element 6 (Adobe Systems, San Jose, CA), where adjustments were made for the whole images for brightness and contrast.

### Reverse transcription and quantitative PCR (RT–qPCR)

Total RNA from FA-D2 fibroblast cells (FA-D2/vec), its corrected cells (FA-D2/D2) and FA-D2 fibroblast cells tranfected with pFlag-FANCD2WT or pFlag- FANCD2KR was isolated with RNeasy (Qiagen). Total RNA was reverse transcribed with an oligo (dT) primer using PrimeScript RT reagent Kit (Takara). Complementary DNA was analyzed in triplicate by qPCR amplification using SYBR Premix Ex Taq II (Takara) with primers to the TNF-α (5′-CATGATCCGGGACGTGGAGC-3′, 5′-CTGATTAGAGAGAGGTCCCTG-3′) or GAPDH (5′-CTCTGCTCCTCCTGTTCGAC-3′, 5′-ACGACCAAATCCGTTGACTCC-3′)}. The PCR amplification conditions were: 95°C (3 s), 42 cycles of 94°C (5 s), 60°C (30 s). Data are presented as fold induction over GAPDH gene and normalized with respect to those obtained in unstimulated FA-D2/vec cells (Figure 3B), or in pFLAG expressing cells (Figure 3D).

### Chromatin immunoprecipitaion (CHIP) assays

FA-D2+Vec cells, FA-D2+D2 cells or HeLa cells transfected with 24 µg of the indicated plasmids (Flag-FANCD2WT, FANCD2KR) using LipofectAMINE 2000 regent (Invitrogen), grown overnight in 100-mm dishes to 60–70% confluency cells. Plates were returned to the incubator for 24 h. At this time, cells were cross-linked with formaldehyde, harvested, and chromatin immunoprecipitations were performed using EZ-ChiIP (Millipore), following the manufacturer's protocol. Each ChIP sample was also subjected to PCR with primers to the distal promoter of TNF-α includingκB1 site (5′-CCACAGCAATGGGTAGGAGAATG-3′, 5′-TTCATGAAGCTCTCACTTCTCAG-3′), the proximal promoter includingκ3 site (5′-GGAGAAGAAACCGAGACAGA-3′, 5′-CTCTGCTGTCCTTGCTGAGGGAG-3′) and the coding region (5′-TCCAGACTTCCTTGAGACAC-3′, 5′-TTGTTCAGCTCCGTTTTCACGG-3′). Antibodies used in the ChIP procedure include antibody to H4-Ac, FLAG as well as rabbit anti-mouse IgG and mouse anti-rabbit IgG. Immunoprecipitated DNA and input DNA were amplified with gene-specific and GAPDH primers (5′-TACTAGCGGTTTTACGGGCG-3′, 5′-TCGAACAGGAGGAGGAGAGAGCGA-3′) by qPCR, using input DNA to generate a standard curve. ChIP data is represented as %input (gene specific)/%input (GAPDH) (Figure 4D), and represented as %input (Figure 4C).

### Purification of human FANCD2

The His_6_-tagged human FANCD2 (hFANCD2) protein was overexpressed in Sf9 insect cells, using the Bac-to-Bac Baculovirus Expression System (Invitrogen). Sf9 cells were infected with recombinant viral particles containing the *hFANCD2* gene, and were cultured for 72 h. The cells were then harvested, and were resuspended in 20 mL TGM-0.5 buffer (20 mM Tris-HCl [pH 8.0], 10% glycerol, 2 mM 2-mercaptoethanol, and 0.5 M NaCl), with Protease Inhibitor Cocktail (Nacalai Tesque). The cells were disrupted by sonication on ice. The supernatant was separated from the debris by centrifugation (27,200 x *g*) at 4°C for 20 min, and was then mixed gently with Ni-NTA agarose beads (6 mL; Qiagen) at 4°C for 1 h. The Ni-NTA beads were washed with 180 mL TGM-0.5 buffer containing 5 mM imidazole, and His_6_-tagged hFANCD2 was eluted with a 100 mL linear gradient of 5 to 400 mM imidazole in TGM-0.5 buffer. The His_6_-tag was removed from hFANCD2 by digestion with thrombin protease (GE Healthcare; 2 U/mg protein) during dialysis against 5 L of TGM-0.2 buffer (20 mM Tris-HCl [pH 8.0], 10% glycerol, 2 mM 2-mercaptoethanol, and 0.2 M NaCl). The protein sample containing hFANCD2 without the His_6_-tag was then loaded onto a Heparin Sepharose CL-6B column (4 mL; GE Healthcare) equilibrated with TGM-0.2 buffer. The column was washed with 80 mL TGM-0.2 buffer, and hFANCD2 was eluted with an 80 mL linear gradient of 0.2 to 1 M NaCl in TGM-0.2 buffer. The fractions containing hFANCD2 were immediately dialyzed against TGM-0.2 buffer (5L). After dialysis, the sample was then loaded onto a Q Sepharose Fast Flow column (4 mL; GE Healthcare) equilibrated with TGM-0.2 buffer. The column was washed with 80 mL TGM-0.2 buffer, and hFANCD2 was eluted with a 40 mL linear gradient of 0.2 to 1 M NaCl in TGM-0.2 buffer. The fractions containing hFANCD2 were immediately dialyzed against TGM-0.2 buffer (5 L). hFANCD2 was loaded on a Mono Q column (1 mL; GE Healthcare) equilibrated with TGM-0.2 buffer. The column was washed with TGM-0.2 buffer (10 mL), and hFANCD2 was eluted with a linear gradient of 0.2 to 1 M NaCl in TGM-0.2 buffer. Purified hFANCD2 was dialyzed against 2 L of TGM-0.2 buffer. The protein concentration was determined by the Bradford method, using bovine serum albumin as the standard protein.

### Nuclear Extracts and Electrophoretic mobility shift assay

HEK293 cells were transiently transfected with 24 µg of the indicated plasmids (empty vector, Flag tagged-FANCD2WT, FANCD2KR, FANCD2KR-Ub) using LipofectAMINE 2000 regent (Invitrogen), following the manufacturer's protocol. Cells were harvested 24 h after transfection and nuclear extracts were prepared as described previously [40]. Samples (200 ng) of recombinant FANCD2 or nuclear extracts (2 mg) were incubated with 32P-labeled NF-κB1 consensus sequence (κB1:5′-GAGTATGGGGACCCCCCCTTAA-3′, κB2:5′-GGGTCTGTGAATTCCCGGGGGT-3′, κ3: 5′-GCTCATGGGTTTCTCCACCAAG-3′) or κB1mutant sequence (κB1 mut:5′-GAGGATCCGGACCCCCCCTTAA-3′) oligonucleotides for 20 min at room temperature in binding buffer [20 mM HEPES at pH 7.6, 5 mM EDTA, 1 mM DTT, 150 mM KCl, 50 mM (NH4)2SO4, and 1% Tween-20 (v/v)]. Following electrophoresis on native 4% polyacrylamide gels, FANCD2-NF-κB1 complexes were visualized by autoradiography.

## Supporting Information

Figure S1 **FANCD2 mutant dose not repress TNF-α-induced NF-κB activity.** **A.** Transiently expressed FANCD2WT repressed enhanced NF-κB activity in FANCD2 deficient cells (PD20:FA-D2). In contrast, FANCD2 monoubiquitination-defective mutant (K561R: D2KR) and FANCC did not change the NF-κB activity. **B**. FANCA deficiency did not significantly enhanced NF-κB activity (p = 0.21114).
(EPS)

Figure S2 **Knockdown of endogeneous p65 represses NF-κB activity.** Cells transfected with the indicated siRNA were treated with TNF-α(20 ng/ml) and NF-κB-luciferase reporter expression was assayed 8 h later.
(EPS)

Figure S3 **EMSA analysis of nuclear extracts isolated from HEK293 cells expressing FANCD2 WT or FANCD2 mutant.** EMSA was conducted using labeled κB1 fragments and 2 µg of nuclear extracts from HEK293 cells transfected with empty vector, Flag-tagged FANCD2 WT, FANCD2 KR, or FANCD2 KR-Ub.
(EPS)
